# A systematic review of methodology used in the development of prediction models for future asthma exacerbation

**DOI:** 10.1186/s12874-020-0913-7

**Published:** 2020-02-05

**Authors:** Joshua Bridge, John D. Blakey, Laura J. Bonnett

**Affiliations:** 1 Department of Eye and Vision, University of Liverpool, Liverpool, UK; 2 Respiratory Medicine, Sir Charles Gairdner Hospital, Perth, Australia; 3 Medical School, Curtin University, Perth, Australia; 4 Department of Biostatistics, University of Liverpool, Liverpool, UK

**Keywords:** Asthma, Exacerbation, Prognostic models, Clinical prediction, Risk, Systematic review

## Abstract

**Background:**

Clinical prediction models are widely used to guide medical advice and therapeutic interventions. Asthma is one of the most common chronic diseases globally and is characterised by acute deteriorations. These exacerbations are largely preventable, so there is interest in using clinical prediction models in this area. The objective of this review was to identify studies which have developed such models, determine whether consistent and appropriate methodology was used and whether statistically reliable prognostic models exist.

**Methods:**

We searched online databases MEDLINE (1948 onwards), CINAHL Plus (1937 onwards), The Cochrane Library, Web of Science (1898 onwards) and ClinicalTrials.gov, using index terms relating to asthma and prognosis. Data was extracted and assessment of quality was based on GRADE and an early version of PROBAST (Prediction study Risk of Bias Assessment Tool). A meta-analysis of the discrimination and calibration measures was carried out to determine overall performance across models.

**Results:**

Ten unique prognostic models were identified. GRADE identified moderate risk of bias in two of the studies, but more detailed quality assessment via PROBAST highlighted that most models were developed using highly selected and small datasets, incompletely recorded predictors and outcomes, and incomplete methodology. None of the identified models modelled recurrent exacerbations, instead favouring either presence/absence of an event, or time to first or specified event. Preferred methodologies were logistic regression and Cox proportional hazards regression.

The overall pooled c-statistic was 0.77 (95% confidence interval 0.73 to 0.80), though individually some models performed no better than chance. The meta-analysis had an I^2^ value of 99.75% indicating a high amount of heterogeneity between studies. The majority of studies were small and did not include internal or external validation, therefore the individual performance measures are likely to be optimistic.

**Conclusions:**

Current prognostic models for asthma exacerbations are heterogeneous in methodology, but reported c-statistics suggest a clinically useful model could be created. Studies were consistent in lacking robust validation and in not modelling serial events. Further research is required with respect to incorporating recurrent events, and to externally validate tools in large representative populations to demonstrate the generalizability of published results.

## Background

Asthma is a complex and heterogeneous syndrome that affects hundreds of millions of people worldwide and has an increasing global prevalence [1]. All individuals with asthma, regardless of age, location or asthma subtype are at risk of a deterioration in symptoms and measures of airway calibre and inflammation that require rescue therapy: an exacerbation. Asthma exacerbations are a common cause of unscheduled healthcare use [1]. In addition to the acute physical morbidity, exacerbations are associated with permanent lung damage [2–4] and have a significant psychological impact [5, 6]. In the United Kingdom, it is estimated that the health service spends £1 billion per year on asthma care with similar indirect costs, particularly from lost work days [7, 8].

Although there has been some improvement in asthma admission and age-adjusted death rates, progress has lagged behind other disease areas. This is despite most asthma exacerbations and deaths being preventable [9]. This slow progress is in part due to the heterogeneity of asthma [10], but also due to the historic focus on daily symptom management. Moving from symptom control to a risk-based strategy has underpinned progress in disease areas such as cardiovascular and diabetic medicine. A method of identifying individuals at a higher risk of asthma exacerbations therefore has appeal [11], particularly where resources are scarce. Generating a personalised risk assessment and targeted management plan in asthma has the potential to significantly improve outcomes.

A prognostic model is a statistical equation that predicts an individual’s outcome risk based on the combination of their values of multiple predictors such as age and sex [12]. Developing a prognostic model generally involves four stages. Firstly, the available data is cleaned and processed. Next the candidate predictors are identified. These are the predictors that are thought to be significant or have previously been linked to the condition of study. The model variables are then chosen from the candidate predictors using multivariable selection methods when possible. Finally, the model performance is assessed, preferably using a different dataset to demonstrate that the model can be extended to new patients [13].

Many papers have studied factors associated with asthma exacerbations. However, these studies have largely considered a narrow group of potential predictors, or have been undertaken in a highly selected population. There have also been prognostic models published that have attempted to address predicting the risk of future exacerbations. The aim of this review was to identify and summarise these prognostic models. Through the identification of existing studies, the review will help to determine whether reliable models have been derived using robust methodology, and what further research is needed within the field.

## Methods

### Search strategy

Five electronic databases were searched in October 2017; four of these were bibliographic and one was a clinical trials register (ClinicalTrials.gov). The bibliographic databases were Medical Literature Analysis and Retrieval System Online (MEDLINE; 1948 onwards), Cumulative Index of Nursing and Allied Health Literature Plus (CINAHL; 1937 onwards), The Cochrane Library, and Web of Science (1898 onwards). All search strategies used both indexed terms and text words to search for asthma and prognostic models [12]. The search strategy is given in Appendix 1. Reference lists of all included papers were checked for potentially contributory papers. There were no language restrictions placed upon articles.

### Inclusion criteria

This review included studies of participants aged 12 years and over, who were diagnosed with asthma and were receiving treatment. The 12 years cut-off for age was chosen based on commonly used criteria in previous asthma studies [14, 15]. Studies focusing on childhood asthma were also excluded from this review. Studies solely concerned with populations with special circumstances (asthma in pregnancy or occupational/ work exacerbated asthma) were excluded, as were those focussed on the assessment of efficacy of a specific trial drug. Papers concerning Asthma-Chronic Obstructive Pulmonary Disorder (COPD) Overlap Syndrome were eligible for potential inclusion given the variation in the use of such terms. Studies that included participants within and outside the inclusion criteria were included if the data of desired participants could be extracted.

Studies deriving prognostic models using multiple factors to predict a clinical outcome of asthma were included. These could be based on randomised controlled trials or observational data. Studies of a single prognostic factor were not considered.

### Study selection

Studies were selected initially from their titles and abstracts, using pre-defined inclusion criteria. A random sub-set (10%) of these studies were reviewed independently by a second reviewer. After initial screening the full texts were obtained, via inter-library and British Library requests where relevant. These full texts were screened with the same pre-determined criteria. Again, a sub-set (10%) of full texts was used in a cross-validation. No discrepancies arose between reviewers in this subset.

The reasons for exclusion were documented and summarised.

### Data extraction

Relevant studies had their data extracted. The pre-piloted data extraction form included the elements pertaining to patient characteristics, statistical modelling and assessment of model performance. Full details are in Appendix 2.

### Quality assessment

To assess the risk of bias (quality) of any included studies both Grading of Recommendations Assessment, Development and Evaluation (GRADE) [16] and the available version of Prediction study Risk of Bias Assessment Tool (PROBAST) were used [17]. GRADE groups studies into outcomes and uses six areas to calculate the quality of evidence. The areas of assessment are study design, risk of bias, inconsistency, indirectness, imprecision, and other considerations such as publication bias. GRADE is specifically intended to evaluate quality of evidence in systematic reviews [16].

The PROBAST tool assesses five domains of bias: participant selection, predictors, outcome, sample size and participant flow, and analysis. Each included study was assessed individually with a risk of bias score (low, high or unclear) being given in each domain. Models were classed as low, moderate or high risk of bias.

### Data synthesis

Each unique model identified was summarised narratively. Information collated included model type, development method, prognostic factors, population characteristics, outcome measures, performance measures (with standard error), and validation methods (internal or external).

A random effects meta-analysis was used to synthesise calibration and discrimination statistics from multiple studies validating the same prognostic model [18]. The meta-analysis was summarised in a forest plot showing the pooled performance. A meta-regression was performed using year of publication and model type as moderators to reduce heterogeneity. Stepwise regression was used to select these moderators, with Corrected Akaike Information Criterion (AICc) used as the criterion [19]. A funnel plot was produced as a visual check for publication bias.

## Results

### Studies identified

**Fig. 1:**
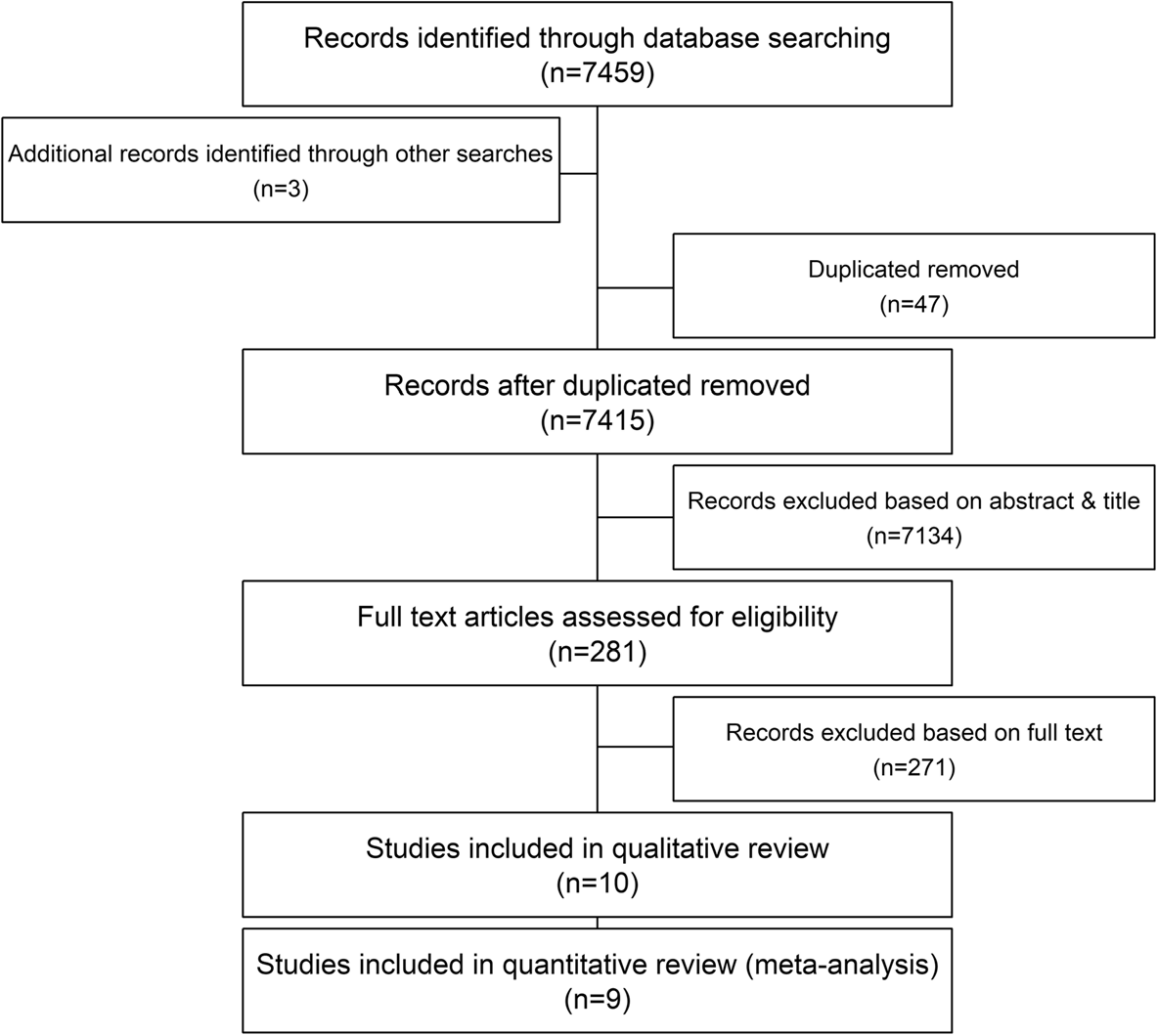
PRISMA flow diagram

The search yielded 7462 results across the five databases, 47 of which were excluded as they were duplicate studies. The remaining titles and abstracts were screened to obtain 281 results, which were deemed to be relevant according to the pre-defined criteria. All studies were published in English. Of these 281 studies, 1 had no full text available via interlibrary loan [20] but the remainder were obtained to further assess their suitability. Using pre-defined selection criteria applied to full texts, 271 studies were excluded. Reasons for exclusion included conference abstract with no related publication (*n* = 31), conference abstract with the full text included elsewhere in the literature search (*n* = 11), letters to the editor (*n* = 18), incorrect study population such as children, pregnant women, or patients with occupational/work exacerbated asthma (*n* = 34), incorrect outcome such as development of asthma, lung function decline, or readmission (*n* = 96), studies of a single prognostic factors (n = 11), and etiological only studies (*n* = 62). This left 13 studies, including 4 on-going studies without relevant publications at the time of the review. Ten studies were included in the qualitative review. One study by Frey et al. [21] proposed a method of predicting future exacerbations using a dynamic model but did not test the method on clinical data. This study was therefore excluded from the quantitative analysis leaving a total of 9 studies [22–30]. A Preferred Reporting Items for Systematic Reviews and Meta-Analyses (PRISMA) flow diagram for this is shown in Fig. 1. Each study is summarised in Table 1.

**Table 1:** Summary of studies included in the quantitative synthesis

Publication	Country	Source	Age of participants	Primary outcome/Definition	Follow up duration	Sample size (Development/Validation)	Events per variable	Predictors in model
Bateman 2014 [22]	Multiple	3 double blind trials Retrospective	12–89	Time to first exacerbation/ GINA	6–12 months	4962/2482	NR/24	Body mass index (BMI), Asthma control questionnaire 5 (ACQ-5) score, Post bronchodilator forced expiratory volume in 1 sec (FEV1), Reliever use, GINA step.
Blakey 2017 [23]	UK	UK electronic medical database Retrospective	12–80	≥2 exacerbations ≥4 exacerbations Asthma related admission or OCS	2 years	118,981	59,514/55 =1082.07	Age, Sex, BMI, Smoking status, Rhinitis, Eczema, Gastroesophageal reflux disease (GERD), Nasal polyps, Anaphylaxis, Non-steroidal anti-inflammatory drug (NSAID) use, Peak expiratory flow (PEF), Blood eosinophil, Mean short-acting beta agonist (SABA) use, Leukotriene receptor antagonist (LTRA) use, Long-acting beta agonist (LABA) use, Inhaled corticosteroid (ICS) use, Acute oral corticosteroid (OCS) use, Asthma related hospital admissions, Primary care consultations.
Finkelstein 2013 [24]	Multiple	Self-reported study Retrospective	Adult	High/low risk of exacerbation/ Patient defined	Not reported	1862/3306	NR/147	Wheeze, Sputum production, Chest tightness, Shortness of breath, Limitation of physical activity, Over-all use of quick-relief inhaler, Exposure to triggers, Over-all asthma estimate, Presence of cold, Number of quick-relief puffs, number of preventative puffs, Sleep problems, Number of times awoken, Last night quick-relief use, Last 24 h prednisone use, Last 24 h nebulizer use, Last 24 h second controller use, Last use of quick-relief inhaler
Finkelstein 2017 [25]	Multiple	Self-reported study Retrospective	Adult	High/low risk of exacerbation/ Patient defined	Not reported	1862/3306	NR/147	Wheeze, Sputum production, Chest tightness, Shortness of breath, Limitation of physical activity, Over-all use of quick-relief inhaler, Exposure to triggers, Over-all asthma estimate, Presence of cold, Number of quick-relief puffs, number of preventative puffs, Sleep problems, Number of times awoken, Last night quick-relief use, Last 24 h prednisone use, Last 24 h nebulizer use, Last 24 h second controller use, Last use of quick-relief inhaler
Honkoop 2013 [26]	New Zealand	Two studies Retrospective	12–75	Severe exacerbation/ Asthma score	1 year	164/94	88/25 =3.52	Asthma symptoms, PEF
Loymans 2016 [27]	Netherlands	ACCURATE study group Retrospective	17–55	Exacerbation ATS/ERS	1 year	611/504	80/15 =5.33	ACQ-5 score, Current smoking status, Chronic sinusitis, Admissions, Oral steroid use, FEV1, Fractional exhaled nitrous oxide (FeNO)
McCarren 1998 [28]	US	Hospital cohort Retrospective	18–55	Severe exacerbation/ Unscheduled ED visit	6 weeks	284	130/18 =7.22	3 or more emergency department (ED) visits in the last 6 months, Difficulty performing activities in the last 4 weeks, Left ED before 24 h without achieving 50% of predicted peak expiratory flow rate (PEFR)
Sato 2009 [29]	Japan	Hospital cohort Prospective	62.5 ± 13.5	Severe exacerbation/ 2 days < 70% PEF	Until event	78	16/4 =4.00	Asthma control test (ACT) score, FEV1, FeNO
Yii 2012 [30]	Singapore	Hospital cohort Prospective	56 ± 18 and 50 ± 19	Severe exacerbation ATS/ERS	3 years	177/84	NR/12	2 or more ED or hospital visits in the past year, BMI, History of near-fatal asthma, Depression, Obstructive sleep apnoea, GERD

*NR* not reported

### Model development methods

There were five distinct types of model development methodology identified in the included studies: probabilistic approaches, logistic regression, survival analysis, action points, and Classification and Regression Tree Analysis (CART).

#### Probabilistic approaches

A probabilistic approach to model building was used in three of the studies [21, 24, 25]. Two of these studies, both by Finkelstein et al. [24, 25], used machine learning to assign a high or low risk of asthma exacerbation for each day, using inputs into a telemonitoring system as predictors. The telemonitoring system used within this study required each patient to have a laptop and connected flowmeter. Both Finkelstein studies aimed to predict an imminent asthma exacerbation, with the 2013 study [25] using a naïve Bayesian classifier and a support vector machine, and the 2017 one [24] adding an adaptive Bayesian network.

Both Finkelstein papers used the same three datasets, with around 70% of the data being used for development and 30% for validation. The accuracies, sensitivities, and specificities of the developed model in each dataset were reported, however these were not split into individual values for development and validation. These machine learning based studies found that an exacerbation could be predicted, using a support vector machine on day eight, with sensitivity 0.84 and specificity 0.80, with the naïve Bayesian classifier showing slightly poorer performance (sensitivity 0.80, specificity 0.77).

The 2017 Finkelstein study again used three datasets with sensitivity and specificity reported for each classifier and dataset, but not distinguishing between development and validation. This study found the adaptive Bayesian network classifier to have higher sensitivity and specificity, than the previous two classifiers.

Frey et al. [21] discussed the use of a complex dynamic model, from statistical physics, to develop a time series based model. This study developed methodology and discussed the theory, however no data was used to produce an actual model, hence no performance measure could be reported. Frey commented that, as well as being used to assess exacerbation risk, this method could be extended to assess exacerbation control and treatment affect.

#### Logistic regression

Three [23, 27, 30] of the studies developed their model using logistic regression. Blakey et al. [23] utilised United Kingdom medical records, resulting in the largest sample size of all the included studies (*n* = 118,981). This dataset was used for both development and internal validation.

Loymans et al. [27] also developed their prognostic model using logistic regression. They used data from the Asthma Control Cost-Utility Randomized Trial Evaluation (ACCURATE) study in model development (*n* = 611) and data from the Unbiased Biomarkers for the Prediction of Respiratory Disease Outcomes (U-BIOPRED) study was used for external validation (*n* = 504). Yii et al. [30] assessed the long-term prognosis of asthma sufferers using logistic regression. They concluded that, over 5 years there are 3 distinct trajectories that sufferers may experience. This study used 177 patients for model development and a validation cohort of 84 patients from the same clinic.

#### Survival analysis

There were two studies [22, 28] developing models using survival analysis. Bateman et al. [22] used three different sets of clinical trial data in an analysis that was not pre-specified. The data were pooled into one set and two-thirds of the data were used for development, whilst the remaining third was used for validation. The risk of severe exacerbation was assessed over 6 months and given values “low” or “high”. McCarren et al. [28] looked at the risk of relapse within 8 weeks of an emergency department visit. No studies considered methods for modelling recurrent events instead modelling time to a specified event.

#### Action points

Honkoop et al. [26] used action points in the self-management of asthma to predict exacerbations. Patients from 3 centres with uncontrolled asthma were included. The study concluded that a composite action point of symptoms and measurements provided the best method of prediction. The optimal action point was found to be an increase of greater than two standard deviations in composite symptom score and a fall in peak expiratory flow greater than 70%, within 1 week of each other.

#### Classification and regression tree analysis

CART analysis was used by Sato et al. [29] A retrospective cohort of 78 patients was used in the development and cross-validation of the model. The CART analysis consisted of three nodes. Similar to the action point study, this study found that a combination of Asthma Control Test and lung function measurements is better at predicting exacerbations than on their own.

## Quality assessment

### GRADE

The GRADE assessment shown in Table 2. One study, using a specific drug as a prognostic factor [22], was funded by a pharmaceutical company and some authors were employees of the company. This study was deemed to be of possible high risk of bias, however the results were consistent with other independent studies.

**Table 2:** GRADE assessment of the included studies

Number of studies	Certainty assessment						Certainty	Importance
	Study design	Risk of bias	Inconsistency	Indirectness	Imprecision	Other considerations		
Exacerbation								
5	Observational studies	Not serious	Not serious	Not serious	Not serious	None	HIGH	CRITICAL
Risk (Categorical)								
2	Observational studies	Not serious	Not serious	Not serious	Not serious	None	LOW	CRITICAL
Time to first severe exacerbation								
1	Observational studies	Not serious	Not serious	Not serious	Not serious	Publication bias strongly suspected dose response curve^a^	HIGH	CRITICAL
Asthma admissions								
1	Observational studies	Not serious	Not serious	Not serious	Not serious	None	HIGH	IMPORTANT
Relapse								
1	Observational studies	Not serious	Not serious	Not serious	Not serious	None	HIGH	IMPORTANT

^a^Contributions from pharmaceutical company employees

### PROBAST

#### Participant selection

Data from observational studies are most informative in prognostic studies, however none of the studies identified used this study design. Models were instead built using data from patients recruited for another purpose (randomised controlled trials, or convenience samples in clinic) and hence highly selected populations. Only McCarron et al. considered people who had initially come to the emergency department [28].

Additionally the studies by Finkelstein et al. used telemonitoring data which may rely on the patients’ ability to use technology. This could potentially exclude certain patients from the study. Three studies [28–30] used data from hospital cohorts which may not be representative of the wider population and may include significant bias. Therefore, overall, we deemed there to be a moderate to high risk of bias from participant selection in most studies.

#### Predictors

All the predictors included in the prognostic models across studies were defined in the same way for each patient within studies. There was incomplete recording of baseline factors in most studies however. One particular cause of concern was in the Finkelstein [24, 25] studies as these rely solely on telemonitoring data which can be highly variable and unreliable [31]. All other predictors were commonly used and shown in previous prognostic factor studies to have evidence of being linked to exacerbations [32]. Some factors such as the Asthma Control Questionnaire score have an inherently subjective nature and rely on the patient scoring themselves. Despite this, the Finkelstein et al. studies [24, 25] were the only trials to be suspected of high bias for predictors – others were deemed to be at a low to moderate risk of bias.

#### Outcome

All included studies used pre-specified outcomes although the outcome was often incompletely recorded. There was only one study, by Honkoop et al., with predictors included in the outcome [26]. This study used two definitions for exacerbations; one definition included symptoms and peak expiratory flow, and these were both also used as predictors. All other studies defined exacerbations independent of predictors.

Asthma exacerbation was defined in a variety of ways across studies with some studies opting for very loose criteria that would be categorised as a deterioration or instability (not an exacerbation). Therefore, for outcome, one study was identified as being at high risk of bias according to PROBAST [26]. The remaining studies were at low to medium risk.

#### Sample size and participant flow

Sample size varied from *n* = 78 [29] to *n* = 118,981 [23] and the number of events per variable (number of asthma exacerbations divided by the number of levels of all candidate predictors) varied from 3.52 to 1082.07 (see Table 1). This ratio was not estimable in four studies which did not report the event rate in the patient group used for model development.

Commonly cited evidence for a minimum number of events per variable is 10 [33]. Assuming a best case scenario (statistically speaking) whereby all patients had an exacerbation, four studies [26–29] failed to achieve the minimum requirement of 10 events per variable which would indicate that these studies were at high risk of bias. Research regarding sample size calculations for external validation studies suggests at least 100 events and 100 non-events are required to access calibration-in-the-large and calibration slope, and at least 200 events and 200 non-events to derive flexible calibration curves for logistic regression [34, 35]. Many of the included studies have not undertaken external validation and where they have the independent data may have insufficient events.

Four [22, 23, 27, 30] of the studies had participants excluded from the final analysis, with two of those not accounting of missing data [27, 30] and hence possibly introducing bias. Some studies used national databases; it is therefore possible that incomplete records were excluded in the searching stage.

Risk of bias was considered to be high in five studies [26–30], moderate in two [24, 25] and low in two [22, 23].

#### Analysis

Throughout the studies, all non-binary predictors and extra complexities were handled appropriately. In particular, univariate model selection was avoided and multivariable model building was undertaken using valid variable selection methods such as backwards selection [36] in all but one study [22].

One study [21] provided no performance measures for its model, while the other studies reported various different performance measures; the c-statistic was the most commonly reported performance measure. Four of the studies [22, 23, 27, 30] either recalibrated or showed evidence that no recalibration was needed through a calibration plot. Two models used bootstrapping in predictor selection [22, 27] and one of these models used bootstrapping in constructing non-symmetric c-statistic confidence intervals [22]. Bias due to the analysis was rated high in five of the studies as these studies used no calibration methods or techniques to avoid overfitting [24–26, 28, 29].

## Meta-analysis

The most commonly reported performance measure was the c-statistic, also called the area under the receiver operating curve [37]. The c-statistic is a measure of discrimination – the diagnostic ability of the model. This statistic was pooled in a meta-analysis to assess the overall effectiveness of the models, with weighting according to sample size. When the model was fit without any moderators, the average overall c-statistic across all models was 0.77 (95% confidence interval 0.73 to 0.80). The pooled c-statistic is above 0.5, the value of chance. From this we can conclude that these models are somewhat effective at predicting the risk of asthma exacerbations.

The Finkelstein studies [24, 25] did not include confidence intervals or standard deviations, and thus it was not possible to construct confidence intervals for these studies. Additionally some studies are very small and thus the estimates of the c-statistic have very wide confidence intervals whilst others have very small confidence intervals suggesting methodological flaws [27, 30].

High variation was found between models, even within the same study. For example, Honkoop et al. [26] report one model using a validation set as having a c-statistic of 0.99 (0.96, 1.00) and the same model and data with a different definition for exacerbation as 0.55 (0.40, 0.70). This is the study identified at being at high risk of bias due to outcome; in definition 1 asthma was defined as an increase in symptoms and peak expiratory flow (PEF), while increase in symptoms and PEF were also the predictors of the model. This will have artificially increased the c-statistic. To test this we removed the models using this definition and refitted the random effects model.

**Fig. 2:**
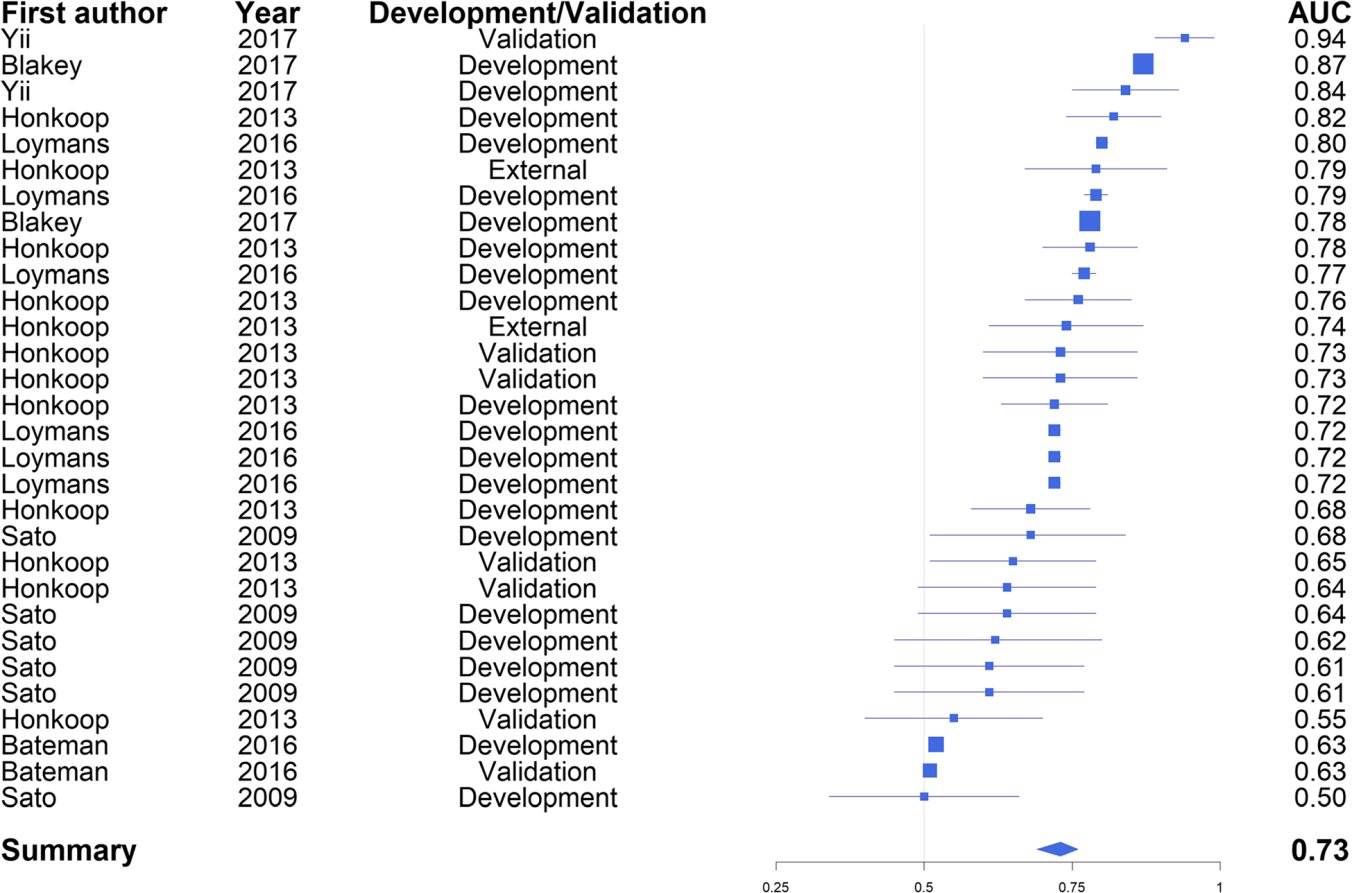
Forest plot of the meta-analysis

**Fig. 3:**
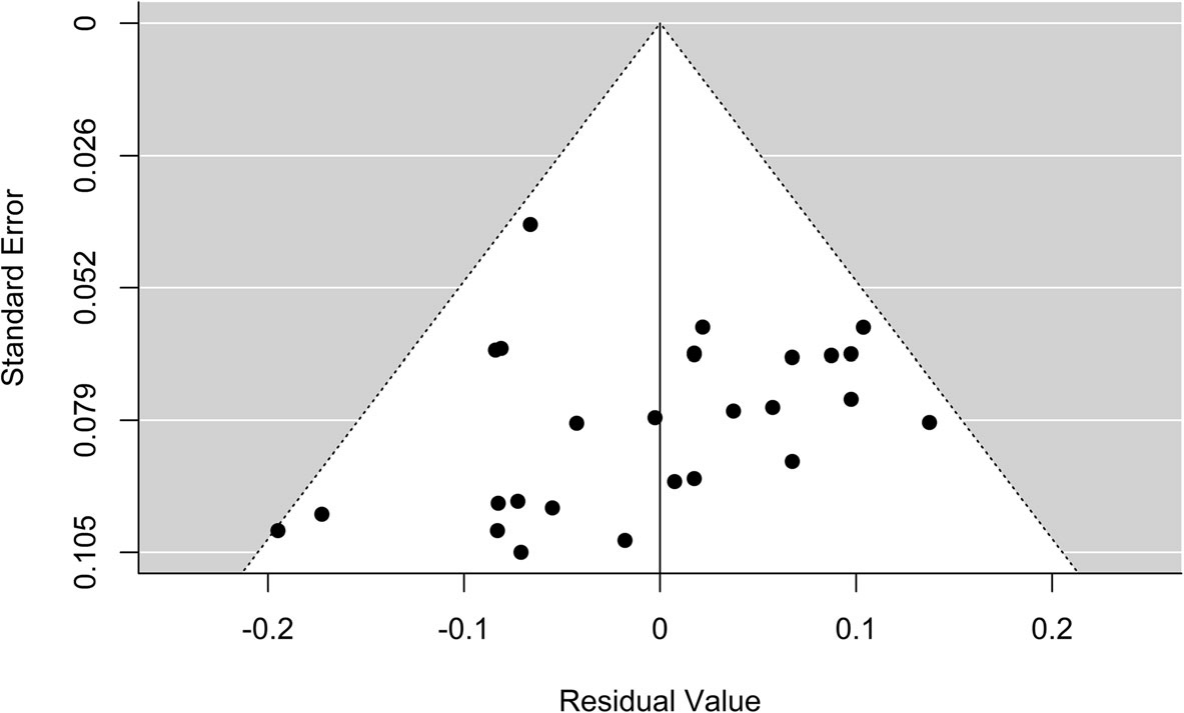
Funnel plot of all models reported in the studies

The forest plot in Fig. 2 shows the results with suspected biased c-statistics removed, with Table 3 displaying the final average c-statistics and confidence intervals. This accounts for 55.7% of the total 99.22% heterogeneity. In Fig. 2 squares are used to depict the c-statistic with associated 95% confidence intervals illustrated by lines. The size of the square is proportional to the sample size. A funnel plot was also produced (Fig. 3), which showed a low risk of publication bias.

**Table 3:** C-statistics with high bias models removed

Selection method	Pooled c-statistic	95% Confidence interval
Logistic regression	0.80	(0.73,0.86)
Optimal action points	0.72	(0.65,0.79)
Other methods	0.62	(0.56,0.67)

The I^2^ statistic was 99.75% demonstrating a large amount of heterogeneity between studies. To reduce heterogeneity, 21 characteristics of the studies, such as recruitment years and model type, were proposed as moderators. This list was derived from the data extraction sheet. The best fitting random-effects model was identified based on AICc by stepwise regression. It was found that the use of logistic regression and optimal action points in the model building were highly significant modifiers. The best random effects model, by AICc, reduced heterogeneity by 42.03%. An additional figure compares the performance of logistic regression models with other models using the development population from each study [see Additional file 1]. This illustrates that newer modelling approaches do not currently perform better than logistic regression.

## Discussion

This systematic review of prognostic models for the risk of asthma exacerbations identified 10 studies developing or validating models. The studies varied in design, study population, statistical approach and definition of outcomes highlighting the need for coordinated programs of research in this area. Three consistent findings were evident from the study. First, from individual study c-statistics and our meta-analyses, it appears possible to create a statistical model for asthma exacerbations which is relatively good at making predictions. Second, study designs using logistic regression or optimal action points had greater accuracy than more complex methods such as survival analysis and CART. Third, far more work has been undertaken in developing models than attempting validation in large, representative populations. The most predictive models tended to include a mix of patient reported outcomes, clinical measurements, and parameters related to medication use.

Our study benefits from a comprehensive search strategy and thorough analysis approach, and is the first such review and meta-analysis in relation to this common and challenging clinical issue. The meta-analysis of heterogeneous studies was undertaken to investigate and demonstrate broad points and therefore the exact c-statistic estimates are not of direct clinical relevance given the degree of study heterogeneity. In particular, the summary statistic from the meta-analysis is to give an indication of the performance, rather than to be an accurate pooled estimate. An additional limitation is the potential for the reviewer bias given that only a random subset of studies were reviewed by a second author. However, no discrepancies between reviewers were identified. Additionally, full text articles were cross-validated, again without disagreements.

The findings of our study are consistent with previous general reviews of clinical prediction models in finding limitations in the design, conduct or reporting of clinical prediction rules research [38, 39]. This lack of robust research is consistent with the development of several prediction models, but none of them being used in routine clinical practice. The situation in asthma is in contrast to other conditions within respiratory medicine such as pneumonia and pulmonary embolism where validated clinical prediction models are commonly used [40]. The success of these tools suggests progress could be made with regard to asthma exacerbations if a coordinated programme of research was undertaken. Our finding that logistic regression appears to be at least as successful for clinical prediction as more complex methods is also consistent with the limited available literature [41], though it fails to account for all the information collected along a patients’ journey such as timing of exacerbations.

Asthma is a very common chronic disease, and acute exacerbations are a major global source of morbidity and both direct and indirect healthcare costs. Providing individuals with a personalised risk assessment has the potential to improve clinical outcomes for those at higher risk, and reduce medication burden for those at lower risk. This has been recognised in the latest international Global Initiative for Asthma (GINA) asthma guidelines [42]. Given the major and increasing pressure on healthcare systems [43] further development of clinical prediction models for asthma appears to be a potentially valuable investment. This may be especially true in lower income countries where asthma is an increasing burden [44] and where risk factors may differ from those identified in North America and Europe [45].

## Conclusion

Our summary of the current state of clinical prediction models for asthma highlights the need for further research at each stage of the development of a tool that would be of clinical use in the future. There is a pressing need for genuinely external validation of existing models in large, well-characterised and representative populations. Consideration must also be given to the best way of addressing the key real world issue of some individuals having multiple events within a specified time frame: their characteristics may be different from individuals that have one episode. There are also many unanswered questions regarding the best way to deploy such prediction tools in clinical practice, and much work to be done in demonstrating both effectiveness and cost-effectiveness as well as optimal presentation method.

### Supplementary information

**Additional file 1.** Forest plot showing the performance of logistic regression vs. other models using the development data from each relevant study.

## Data Availability

The datasets created during the current study are available from the corresponding author on reasonable request.

## Appendix 1

### Medline search strategy example (Adapted from Ensor et al. [17])

1. Risk Assessment/ or Models, Statistical/ or (predict* or validat* or rule* or scor*).ti,ab. or ((predict* or multicomponent or multivariable) adj3 model*).mp. or (predict* adj5 (outcome* or risk* or model*)).ti,ab. or ((history or variable* or criteria or scor* or characteristic* or finding* or factor* or value*) adj5 (predict* or model* or decision* or identif* or prognos*)).ti,ab. or (decision* adj5 (model* or clinical* or logistic model*)).ti,ab. or (prognostic adj5 (history or variable* or criteria or scor* or characteristic* or finding* or factor* or model*)).ti,ab. or (observ* adj3 (variation or model*)).ti,ab.

2. asthm*.tw. and (exacerbat* or attack*).mp.

3. 1 and 2

## Appendix 2

### Data extraction

The data extraction form included the following elements:

- Article information (e.g. Author, Title, Year of Publication)
- Study information (e.g. Country, Sample size, Recruitment dates)
- Study design characteristics (e.g. design, length of follow-up)
- Patient characteristics (e.g. Ages, Sexes, Asthma diagnosis)
- Predictors (Candidate, Final, Continuous/Dichotomous)
- Statistical methods (Predictor selection, Fitting)
- Models (Development/Validation, Risk Measure)
- Model performance measures (Area under the receiver operating curve)

## Abbreviations

ACCURATE: Asthma Control Cost-Utility Randomized Trial Evaluation
AICc: Corrected Akaike’s Information Criterion
CART: Classification And Regression Tree analysis
CINAHL: Cumulative Index of Nursing and Allied Health Literature
COPD: Chronic Obstructive Pulmonary Disorder
GINA: Global Initiative for Asthma
GRADE: Grading of Recommendations Assessment, Development and Evaluation
MEDLINE: Medical Literature Analysis and Retrieval System Online
PEF: Peak Expiratory Flow
PRISMA: Preferred Reporting Items for Systematic Reviews and Meta-Analyses
PROBAST: Prediction Study Risk of Bias Assessment Tool
U-BIOPRED: Unbiased Biomarkers for the Prediction of Respiratory Disease Outcomes
