# Supplementary material for: A systematic review of methodology used in the development of prediction models for future asthma exacerbation
Source: BMC Med Res Methodol. 2020 Feb 5;20:22. doi: 10.1186/s12874-020-0913-7 (PMC7003428; doi:10.1186/s12874-020-0913-7)

Additional File 1

Additional Figure 1: Forest plot showing the performance of logistic regression vs. other models using the development data from each relevant study

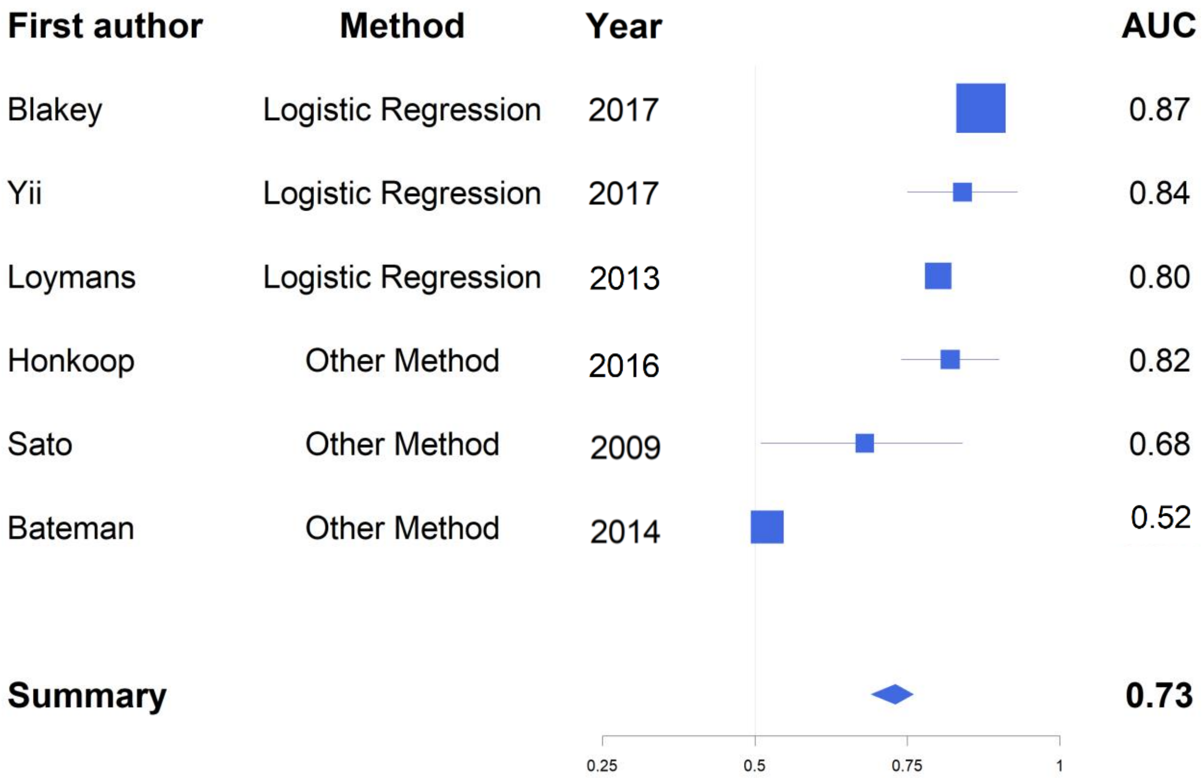

Supplement: Supplementary file 1 — Additional file 1. Forest plot showing the performance of logistic regression vs. other models using the development data from each relevant study. [file 12874_2020_913_MOESM1_ESM.pdf]
